# Impact of the COVID-19 pandemic on utilization and cost for care of pediatric and young adult ALL

**DOI:** 10.1186/s13104-024-06768-x

**Published:** 2024-04-22

**Authors:** Alex Hoover, Dave Watson, Paige Reimche, Lynn Tanner, Laura Gilchrist, Mike Finch, Yoav H Messinger, Lucie M. Turcotte

**Affiliations:** 1 Optum Labs Visiting Fellow, Division of Pediatric Blood and Marrow Transplantation, University of Minnesota Medical School, Mayo Mail Code 366, 420 Delaware St SE, 55455 Minneapolis, MN USA; 2 Children’s Minnesota, Minneapolis, MN USA; 3 St. Catherine University, St. Paul, MN USA

**Keywords:** Pediatric, Young adult, Acute Lymphoblastic Leukemia (ALL), Care, Cost, Utilization, COVID-19, Pandemic

## Abstract

**Objective:**

Acute lymphoblastic leukemia (ALL) is the most common childhood malignancy and among the most common malignancies in young adults and requires a unique pattern of healthcare utilization including an acute/emergent presentation and an intensive initial 8 months of therapy followed by two years of outpatient treatment. The COVID-19 pandemic caused massive global disruptions in healthcare use and delivery. This report aims to examine the effects of the COVID-19 pandemic on the presentation, diagnosis and continued management of childhood and young adult ALL in regard to utilization and cost of care among commercially insured individuals in the United States.

**Results:**

Utilizing a commercial insurance claims database, 529 pediatric and young adult patients were identified who were diagnosed with ALL between January 2016 and March 2021. New diagnoses were evaluated by era and demographics. Utilization was measured by COVID-related era as number of inpatient and outpatient encounters, inpatient days, and cumulative cost during the initial 8 months of therapy. None of these cost or utilization factors changed significantly during or shortly after the pandemic. These findings reinforce that the necessary care for pediatric and young adult ALL was unwavering despite the massive shifts in the healthcare system caused by the COVID-19 pandemic. This provides a valuable benchmark as we further examine the factors that influence the pandemic’s impact on health equity and access to care, especially in vulnerable pediatric and young adult populations. This is the first investigation of the effect of the COVID-19 pandemic on utilization and cost of care in pediatric and young adult cancer.

## Introduction

Acute lymphoblastic leukemia (ALL) is the most common cancer of childhood and adolescence, with approximately 3000 new cases diagnosed each year in individuals under age 18 years of age in the United States (US) [1]. Although the incidence decreases with age, it remains among the most common malignancies for young adults as well [1]. The COVID-19 pandemic affected healthcare use and delivery across the United States and internationally, with multiple studies showing decreased healthcare utilization in both the emergency and ambulatory setting, particularly in the early months of the pandemic [2–4]. A reduction in visits due to patient fear of infection and reduced access to typical care due to public health regulations led to dramatic reductions in the use of preventive and elective care, including cancer-related care, during the first and second quarter of 2020 [5]. Pediatric and young adult ALL requires unique healthcare utilization including elements of acute/emergent presentation, an intensive initial 6–8 months of treatment and nearly two years of lower intensity outpatient treatment. We sought to examine the effects of the COVID-19 pandemic on the presentation, diagnosis and continued management of childhood ALL in regard to utilization and cost of healthcare and to examine whether changes in use and cost differ by patient characteristics.

## Methods

Using de-identified commercial insurance data from the OptumLabs® Data Warehouse, a cohort of patients with ALL was identified, aged 1–30 years and diagnosed between January 2016 and March 2021 in the United States. The ages for inclusion were selected based on inclusion criteria for previous Children’s Oncology Group ALL treatment protocols, which included individuals aged 1–30 years. Notably, the most recent ALL protocol (AALL1732), which opened in June 2019, included individuals aged 1–25 years; however, many individuals treated for ALL as young adults are treated based on pediatric ALL protocols. Date of ALL diagnosis was confirmed based on initial ICD-9 and ICD-10 diagnostic codes (204.00, 204.01, 204.02 and C91.00, C91.01, C91.02, respectively) in combination with either a CPT code for bone marrow biopsy or lumbar puncture within 14 days of the first ALL diagnostic code. Total number of new diagnoses were identified and stratified by timing of diagnosis, including early pre-COVID era (1/2016–6/2019), overlap COVID era (7/2019–3/2020), and COVID era (4/2020–3/2021). New diagnoses were also evaluated by age, sex and region of the US for differences in diagnostic patterns. Utilization was measured as number of inpatient and outpatient encounters, inpatient days, and cumulative cost (inflation adjusted) for the initial 8 months of therapy. Associations of demographics and utilization outcomes with era of diagnosis were assessed using chi-square test (or Fisher exact test when necessary) and Kruskal-Wallis test. R was used for statistical analyses [6]. 

## Results

Among the 529 identified pediatric and young adult ALL patients diagnosed within the predetermined timeframe, 42.5% were female and median age at diagnosis was 7 years (interquartile range 4–15 years). There were 363 new ALL diagnoses in the pre-COVID era, 63 in the overlap COVID era, and 103 in the COVID era. These frequencies were proportional to the length of the respective eras with 8.6, 7.0, and 8.6 diagnoses per month, respectively (goodness-of-fit chi-square *p* = 0.29). Patient characteristics of new ALL diagnoses across all three eras were similar with respect to age, sex, and geographic region (Table 1).

**Table 1:** Patient characteristics and utilization measures by era in which ALL diagnosis occurred

Characteristic		Overall *N* = 529	Pre-COVID *N* = 363	Overlap COVID *N* = 63	COVID era *N* = 103	*p*-value*
Median age, years (Q1, Q3)		7 (4, 15)	7 (4, 16)	8 (4, 17)	7 (4, 14)	0.72
Sex, n (%)	Female	225 (42.5)	148 (40.8)	30 (47.6)	47 (45.6)	0.46
	Male	304 (57.5)	215 (59.2)	33 (52.4)	56 (54.4)	
Region, n (%)	Midwest	149 (28.2)	100 (27.5)	18 (28.6)	31 (30.1)	0.62
	Northeast**					
	South	219 (41.4)	148 (40.8)	29 (46)	42 (40.8)	
	Unknown**					
	West	98 (18.5)	74 (20.4)	7 (11.1)	17 (16.5)	
Median outpatient encounters (Q1, Q3)		64 (51, 81)	64 (51, 82)	59 (50, 82.5)	64 (50.5, 76.5)	0.62
Median inpatient encounters (Q1, Q3)		5 (3, 7)	6 (3, 8)	5 (2.5, 7)	5 (2, 7)	0.48
Median inpatient days (Q1, Q3)		38 (21, 58)	38 (23, 58)	37 (19, 59.5)	41 (18, 56)	0.84
Median costs, $1000 (Q1, Q3)		495 (282, 783)	491 (286, 797)	501 (298, 690)	560 (257, 770)	0.92

*Chi-square test was used for sex, Fisher’s exact test was used for region (including censored cells), and Kruskal-Wallis test was used for remaining numeric characteristics

**Blank cells correspond to censored cells; multiple cells need to be blanked so frequencies cannot be recovered, per OptumLabs® guidelines

**Fig. 1:**
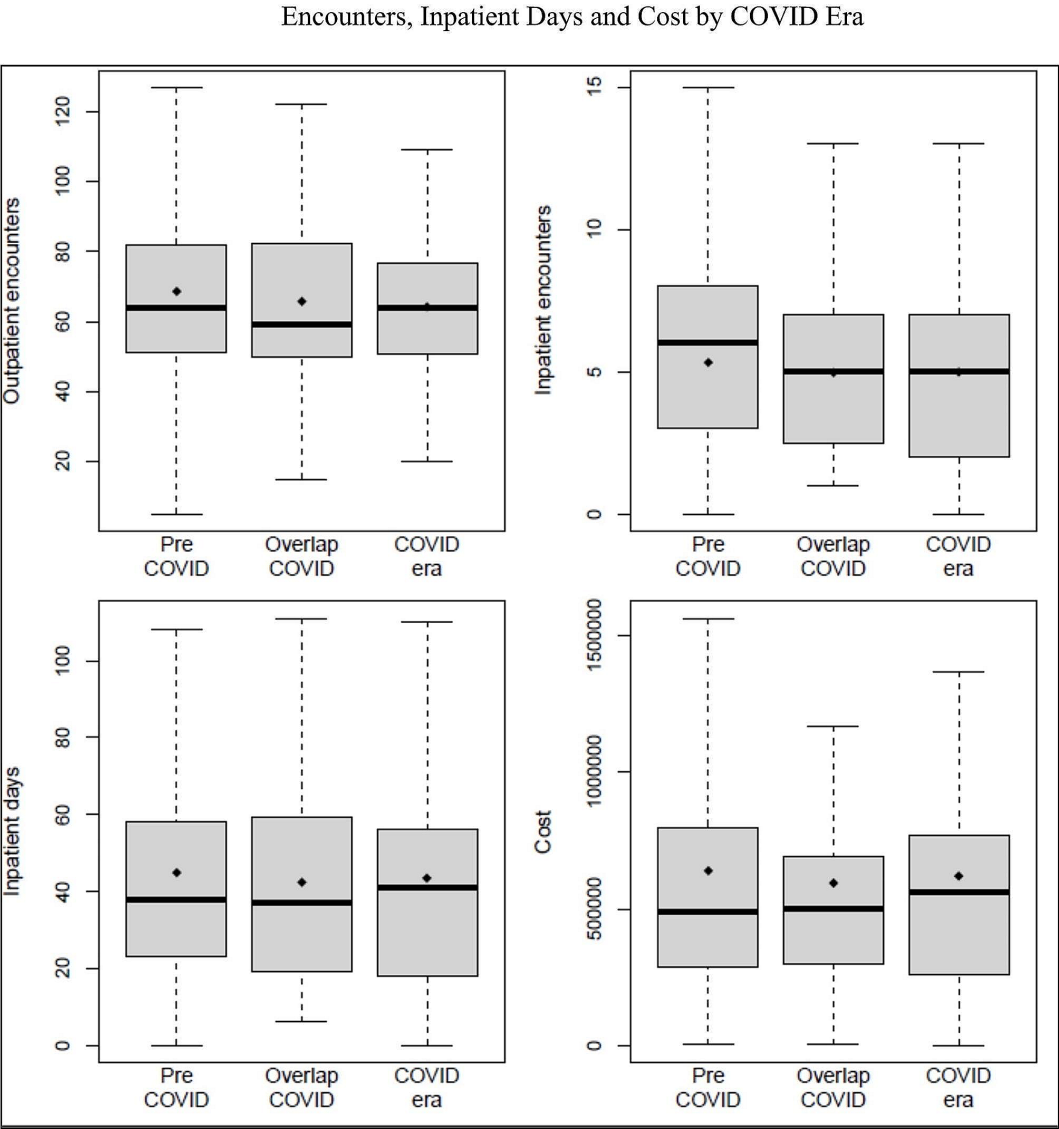
Inpatient/outpatient encounters, inpatient days and cost by COVID era (Pre-COVID era (1/2016–6/2019), overlap COVID era (7/2019–3/2020), and COVID era (4/2020–3/2021))

Utilization for all four measures were similar across all three eras (Figure 1). Over the first 8 months of therapy, the median utilization was 5 inpatient encounters, 64 outpatient encounters, 38 total inpatient days, and $495,000 for cumulative cost of care; these values did not vary significantly by era (all *p* > 0.05, Table 1).

## Discussion

This real-world cost analysis shows for the first time the effect of the COVID-19 pandemic on patterns of care for pediatric and young adult ALL– encompassing not only the pattern of new diagnoses but also patterns of care and cost during the initial 8 months of intensive therapy. We show that the number of inpatient and outpatient encounters, inpatient days and cost of care did not change significantly during the pandemic, reinforcing the fact that the care for pediatric and young adult ALL was unwavering despite the massive shifts in the healthcare system caused by the COVID-19 pandemic. Although the median cost increased between the pre-COVID and COVID eras, the change was not statistically significant and variability in cost was relatively large, consistent with our previous report [7]. 

Investigations into pandemic-related changes in care for other pediatric diseases that require significant medical care and attention at diagnosis include diabetic ketoacidosis (DKA) for new-onset type 1 diabetes have found an increase in incidence of DKA and severe DKA [8]. Shifts in access to primary care and patient fear of infection with accessing the healthcare system have been attributed to these alterations in diabetes presentation and DKA severity [9]. 

However, in the face of major decreases in preventative and elective healthcare shown in other studies, we have shown that care for pediatric and young adult ALL continued at a steady rate throughout the early pandemic time period. This study provides a valuable benchmark of patterns that can be reassessed over time as we attempt to further examine the factors that influence the pandemic’s impact on health equity and access to care, especially in vulnerable pediatric and young adult populations.

## Limitations

Despite the many strengths of this study, limitations must be acknowledged. The use of claims data relies on accurate and consistent coding, which can make it difficult to ascertain the exact date of diagnosis or relapse. We were unable to comprehensively evaluate racial or ethnic differences in pandemic-era ALL cost or care utilization given the large proportion of individuals with missing or undefined race and ethnicity data. Additionally, the OptumLabs Data Warehouse is limited to commercially insured individuals, thus excluding publicly insured or managed care patients from this analysis and limiting the generalizability of these results.

## Data Availability

The dataset supporting the conclusions of this article is available in the OptumLabs data repository, with restricted access but available upon request.

## Abbreviations

ALL: Acute lymphoblastic leukemia
DKA: Diabetic ketoacidosis
ICD: International statistical Classification of Diseases
US: United States
